# The effect of mobile-based logotherapy on depression, suicidal ideation, and hopelessness in patients with major depressive disorder: a mixed-methods study

**DOI:** 10.1038/s41598-023-43051-8

**Published:** 2023-09-22

**Authors:** Maryam Shaygan, Fahimeh Alsadat Hosseini, Marzieh Shemiran, Arvin Hedayati

**Affiliations:** 1 Community Based Psychiatric Care Research Center, School of Nursing and Midwifery, Shiraz University of Medical Sciences, Shiraz, Iran; 2 Student Research Committee, Department of Psychiatric Nursing, School of Nursing and Midwifery, Shiraz University of Medical Sciences, Shiraz, Iran; 3 Research Center for Psychiatry and Behavior Science, School of Medicine, Shiraz University of Medical Sciences, Shiraz, Iran

**Keywords:** Psychology, Diseases, Health care

## Abstract

Major depressive disorder is one of the most common psychiatric disorders in the world. It is essential to study and use effective, available, and affordable psychotherapy methods along with drug therapy to manage the symptoms of this disease. Therefore, the current study aimed to determine the effect of mobile phone-based logotherapy on depression, suicidal ideation, and hopelessness in patients with major depressive disorder by using a mixed-methods approach. In the first phase of this mixed-methods study, 70 patients completed the quantitative phase (control group = 35, intervention group = 35). The intervention group received an 8-week mobile-based logotherapy program via WhatsApp (one 180-min module per week) combined with sertraline, while the control group received just sertraline plus education about pharmacotherapy. Data was collected before, immediately after the intervention, and 3 months later using the Beck depression inventory short form items (BDI-13), the Beck hopelessness scale (BHS), and the Beck scale for suicide ideation (BSSI). Then, a qualitative study on the intervention group was conducted to explain the findings of the quantitative phase. The repeated measure MANOVA revealed a significant interaction effect of time and group on the set of dependent variables (F(6,63) = 25.218, P < 0.001). Qualitative analysis confirmed the efficacy of sertraline plus mobile-based logotherapy on depression, suicidal ideation, and hopelessness in the intervention group. Three key themes extracted from the participants’ experiences of mobile-based logotherapy were “efficient instruction”, “user-friendly intervention” and “constructive change”. Mobile-based logotherapy through WhatsApp was an effective psychotherapy method for decreasing depression, hopelessness, and suicidal ideation in patients with major depressive disorder. It is suggested that educational, institutional, and technological infrastructure for providing and using mobile-based logotheapy for patients with major depressive disorder be considered in the mental health care system.

## Introduction

Depression is one of the most common psychiatric disorders in the world. This disease is still an important issue in public health^1^. The prevalence of depressive symptoms has increased by 34% from 2001 to 2020^2^. Also, recent studies have shown that the epidemic of COVID-19 has had a great impact on people’s mental health and has increased the prevalence of depression^3,4^. Depression is one of the problems that greatly hinders the growth, activity, and development of young people. It is also considered a risk factor for diseases such as cancer and heart disease^5,6^. On the other hand, it also imposes heavy financial costs on society in the treatment sector^7^.

Major depressive disorder (MDD) is associated with symptoms such as depressed mood, loss of interest and motivation, hopelessness, lack of enjoyment in activities, and decreased energy. These symptoms cause a decrease in individual and social performance in people^8^. Also, people with MDD have higher levels of suicidal thoughts, suicide attempts, and suicide-related deaths than people without this diagnosis^9,10^. Hopelessness, defined as negative expectations about the future, is the link between depression and suicide^11^. The theory of depression known as the “hopelessness theory” suggests that feelings of hopelessness can directly lead to the development of depression^12^. When experiencing intense hopelessness, people believe that there is no end to their feelings and no solution for them. Previous studies show the importance of paying attention to the feeling of hopelessness as a warning sign of the onset of depression and suicide attempts^13–15^. Therefore, treating the symptoms of hopelessness, depression, and suicidal ideation is very important.

Through previous studies, it has been determined that sertraline may be one of the best options for the treatment of moderate to severe MDD^16^. In addition, some studies have shown that adding psychotherapy to antidepressants may have stronger effects on reducing depressive symptoms^17,18^. Psychotherapists use different approaches to treat the symptoms of depression and hopelessness. One of the therapeutic approaches that has recently attracted the attention of researchers in the field of depression is logotherapy. According to Viktor Frankl, logotherapy is “treatment through meaning”^19^.

Frankl’s theory suggests that discovering meaning in life is a fundamental human aspiration and a motivating force that helps individuals attain emotional and spiritual welfare^20^. Meaning therapy enables a person to discover the unique meaning of his life in three ways: paying attention to creative values, paying attention to experiential values, and paying attention to attitudinal values. Creative values are doing something valuable for others. Experiential values involve experiencing something or someone that a person considers valuable. The most important example of experiential values is enjoying nature, being kind, and loving welfare^20^. Attitudinal values include virtues such as compassion, courage, and humor, and the most important example of attitudinal values is finding meaning through suffering and welfare^20^. Meaning therapy states that what makes a person weak and defeated is not suffering and hardships but the meaninglessness of life, and if suffering is bravely accepted, life will find meaning. Therefore, in this approach, people are encouraged to discover the meaning of their sufferings and welfare^20^.

Most of the recent research shows that helping to find meaning and purpose in life using the logotherapy approach may have positive effects such as reducing the symptoms of depression, hopelessness, and suicidal thoughts and improving the quality of life for a person. However, most of these studies have been conducted on patients with cancer^21,22^, substance abuse^23,24^, diabetes^24^, students^25,26^ and older adults^27^. For example, the systematic review study by Koulaee et al. showed that logotherapy has a significant role in reducing depression in cancer patients^28^. Also, two recent quasi-experimental studies have shown the effectiveness of logotherapy in reducing depression in patients with depression admitted to the psychiatric ward^29^ and older adults with depression^30^. Some studies have also investigated the effect of legotherapy on hope in patients with depression^31^ or leukemia^32^. Based on the results of the quasi-experimental study of Sun et al., logotherapy was an effective method for reducing suicidal ideation in patients with depression^29^. However, knowledge in this area is limited by the lack of randomized controlled trials (RCT)^29,31^, the lack of control for antidepressant treatment^29,31,33,34^, mostly including patients with cancer^29,35^, mild depression^31^ and a very small sample size^25,30,31^. To our knowledge, no logotherapy-based RCT specifically targeting depressive symptoms in outpatients with major depressive disorder has yet been implemented. Also, there are some conflicting results in the field of logotherapy’s impact on the various variables^29,36,37^. In addition to the limitations mentioned in previous studies, it is important to note that it is not possible to benefit from face-to-face psychotherapy for all patients. Some patients live in remote areas far from big cities. Therefore, due to both the distance and the high costs of psychotherapy, especially in countries where psychological services are not covered by insurance, it is not possible for many patients to utilize many modern psychotherapy approaches, including logotherapy. Some studies indicate that providing mental health services through the Internet and related technologies is a useful and constructive approach in the treatment of depression^38,39^. Therefore, remote psychotherapy may be a promising way to treat depression in conditions where mental health service resources are limited.

Research is required to identify the psychological interventions that can be used to reduce depression and hopelessness levels and suicidal ideation and to determine whether the goals of psychotherapy approaches could be accomplished with mobile-based interventions that could be widely distributed and self-administered. Given the novel opportunities and recognition of the rapidly expanding field of digital health research^40^, it seems worthwhile to provide the first randomized controlled trial on mobile-based logotherapy for depression^38^. As such, the current study aims to investigate mobile-based logotherapy regarding its feasibility, adherence, patient satisfaction, and effectiveness on depressive symptoms, hopelessness, and suicidal ideation in outpatients with moderate or severe MDD while controlling for antidepressant medication. It is assumed that the patients receiving mobile-based logotherapy would report decreased hopelessness (the primary outcome) and consequently decreased depressive symptoms and suicidal ideation (the secondary outcomes) compared with those in the control group.

## Methods

### Study design

To gain a more comprehensive understanding of the quantitative results, we utilized a mixed-methods sequential explanatory design that involved two phases. In the first phase, we collected and analyzed quantitative data through anonymous online surveys. In the second phase, we collected qualitative data through telephone interviews, which were then analyzed. This approach, which incorporated both quantitative and qualitative techniques, aimed to provide a more detailed interpretation of our research findings.

In our study, we assigned a slightly higher weight to quantitative data. We used the quantitative findings to guide purposeful sampling and refine interview questions during the qualitative phase. In our study, we integrated the results of both the quantitative and qualitative phases during the discussion of the findings. By combining the two types of data, we aimed to gain a comprehensive understanding of the factors that contribute to the effectiveness of mobile-based logotherapy on hopelessness, depression, and suicidal ideation in patients with major depression. These findings could then be used to guide the development of mobile-based psychotherapy, with a specific focus on logotherapy.

### Quantitative phase

The quantitative phase of our study was a randomized, controlled, parallel-group clinical trial with a 1:1 ratio. We followed the CONSORT 2010 guidelines^41^ in performing and reporting all methods used in the trial.

### Participants and settings

Our study sample consisted of adult patients referred to three outpatient psychiatric services located in the southern region of Iran. All participants had a primary diagnosis of unipolar MDD, which was defined according to the *Diagnostic and Statistical Manual of Mental Disorders*, *Fifth Edition (DSM-V)*^42^ and confirmed by a psychiatrist using the Structured Clinical Interview for DSM (SCID-5). To be eligible for participation, the following inclusion criteria were employed: the participant had to be over 18 years old, willing to take part in the study, taking sertraline as their antidepressant medication, free of other antidepressants (except sertraline) for at least two weeks prior to the study, have at least moderate to severe depression (scores ≥ 10) according to the Beck Depression Inventory short form (BDI-SF)^43^, a Beck Hopelessness Scale rating of ≥ 9 indicating at least moderate severity of hopelessness^44^, have internet access, and be able to work with the media. The following exclusion criteria were applied: a history of bipolar disorder, the presence of psychotic symptoms, a diagnosis of severe psychiatric disorders necessitating hospitalization, severe medical or psychiatric illness interfering with psychotherapy, a suicide risk requiring immediate hospitalization, receiving other medications or treatments besides sertraline and logotherapy during the study, experiencing a stressful event recently or during the study period, and reluctance to continue contributing to the study.

### Randomization and blinding

Patients were randomly assigned to two groups: logotherapy plus sertraline (the intervention group) and sertraline plus education about drug therapy with sertraline (the control group). Randomization was done using a computerized randomization program. In order to blind and prevent possible bias, dividing the groups into two groups, collecting data, interpreting questionnaires, and entering and analyzing data in statistical software were all done by the researchers' assistants and statisticians outside the study. Although, due to ethical principles, all patients were aware of the subject under study (logotherapy), they were not aware of the exact content of the trainings in the other group and did not know what intervention the patients in the other group received.

### Treatment conditions

All patients in the present study were treated with sertraline. A flexible-dose design was used. The dose was being adjusted based on clinical response and tolerability. For all patients, treatment was started at a dose of 25 mg/day. After one week, depending on the clinical response and side effects, the dose was increased to 50 mg/day. After that, in the absence of dose-limiting side effects, the drug dose could be flexibly increased up to a maximum of 200 mg per day until a satisfactory clinical response is reached^45^. A satisfactory clinical response to sertraline treatment was considered to be at least a 50% reduction in the BDI-SF baseline score during 8 weeks^46^. Patients could be withdrawn from the study at any time for reasons such as side effects or insufficient therapeutic response despite increasing the sertraline dose.

### Intervention condition: sertraline plus mobile-based logotherapy

The intervention condition in this study involved participants receiving mobile-based logotherapy in combination with pharmacotherapy using sertraline over an 8-week period. The mobile-based logotherapy consisted of 8 weekly modules based on Frankl’s meaning-centered psychotherapy^20^, which is a suitable approach for enhancing psychological resilience during life transitions. Participants were instructed to complete one module per week, with each module designed to take approximately 180 min. The modules included videos, audio files, educational texts, and one or two exercises related to the module’s content, which were designed by a team of psychologists and psychiatric nurses supervised by the first author. WhatsApp was used to deliver educational modules (videos, podcasts, and educational texts) to the patients 3 days a week between 9 and 10 AM. The content of the modules is presented in Table 1. The patients were encouraged to study the modules daily, practice the exercises regularly, and participate in group discussions (through WhatsApp groups) to provide feedback on the modules and exercises. Regular contact was maintained with the patients through text messages and/or phone calls to ensure they were using the modules and practicing the exercises appropriately.

**Table 1:** Topics of modules/session.

Module/week	Educational packages	Contents
1	Educational package 1	Expressing the goals and plans of the group and getting to know Frankel’s life Expressing the causes of depression and despair based on the logotherapy approach and self-awareness education Conducting group exercises with the aim of increasing awareness of one's strengths and weaknesses, realized and unrealized wishes and desires, hopes, and expectations The participants were asked to answer these questions: “Who am I? Who do I want to be? What do I like, and what do I dislike?”
2	Educational package 2	Getting to know the ways of achieving meaning in life Getting to know the triple values from Dr. Frankel's point of view Explaining the stages of determining life values The participants were asked to adjust their life goals and values In the group exercise, the participants were asked to imagine themselves instead of the person they wanted to be like
3	Educational package 3	Getting to know the meaning of life and nature Getting to know love and the meaning of life Getting to know the methods of self-love and gratitude Participants were asked to do exercises of love and gratitude Participants were asked to make a list of things that deserve gratitude
4	Educational package 4	Getting to know creative ways and enjoying doing work Expressing the importance of hope in life In the group exercise, the participants were asked to do a useful task or valuable activity for themselves or others this week and share it with the group members Also, in order to overcome limiting thoughts, the participants were asked to answer the question, “If God were to fulfill your three wishes, what would you wish for?”
5	Educational package 5	Getting to know the meaning of life and moral virtues Getting to know the ways of finding the meaning of life in the suffering and limitations of life The participants were asked to describe the meaning of a particular situation in their life with the aim of focusing on the long-term meaning of life In a group exercise, the participants were asked to choose the words of distinguished persons about moral virtues, hope, and suffering in life and share them with the group members
6	educational package 6	Getting familiar with the concepts of death and loneliness and finding the meaning of life in different aspects of life Teaching positive thinking techniques The participants were asked to use these techniques in necessary situations and share the results with the group members
7	Educational package 7	Expressing the roles and remembering the responsibilities of the current life Expressing the concepts of freedom and authority, responsibility, and effort Teaching anger management techniques and effective communication The participants were asked to draw their determined values and meanings on the path of a mountain towards the top, with the aim of setting goals and accepting responsibility and autonomy
8	Educational package 8	Familiarity with logotherapy techniques, including methods such as conflicting intentions, de-reflection, laughter, and the use of humor to address fears and problems The participants were asked to practice laughing in front of the mirror With the aim of improving spiritual skills, mindfulness exercises were taught In a group exercise, the participants were asked to set valid goals for their lives The participants were asked to consider a personal contract between themselves and God with their trust from the heart in God

### Control condition: sertraline plus education about pharmacotherapy

In addition to drug therapy, the participants in the control group received drug therapy training with sertraline. The educational content included videos and educational literature, totaling 40 min, about the drug sertraline, which was designed by a team of psychiatrists and psychiatric nurses under the supervision of the first author. Educational contents were provided 1 day a week between 9 and 10 a.m. through WhatsApp.

### Recruitment and procedure

Patient recruitment was conducted between February 20 and March 20, 2022. First, the characteristics of 103 patients who had unipolar MDD according to the psychiatrist’s diagnosis and according to DSM-V criteria and were prescribed sertraline were given to the research team.

**Figure 1:**
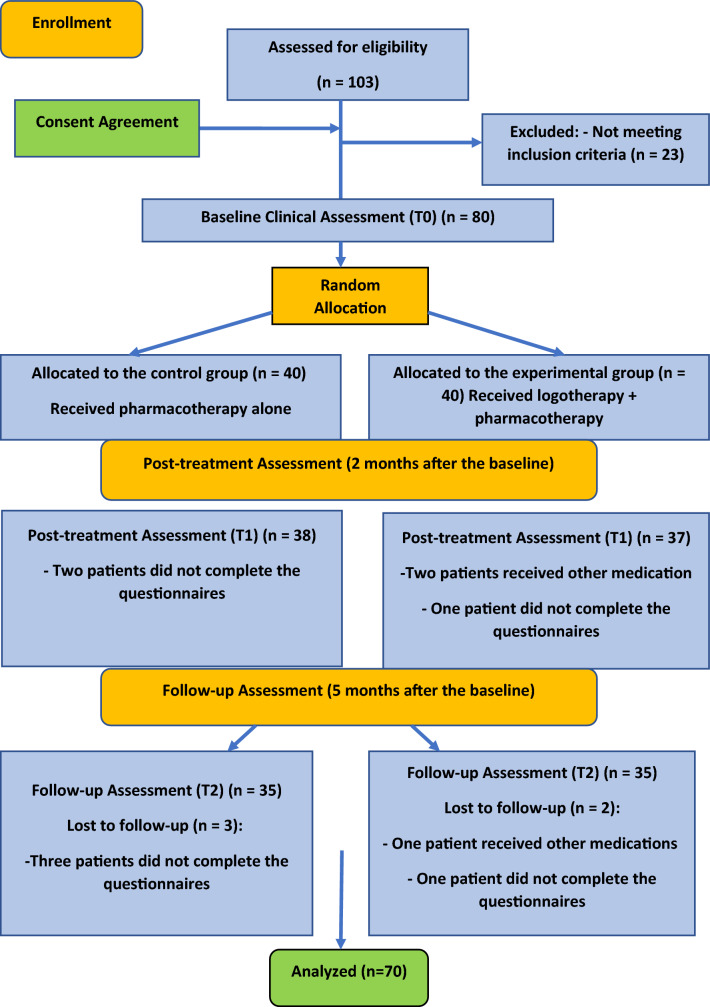
CONSORT flow diagram.

Then, a psychiatric nurse (the third author) contacted the patients by telephone, informed them about the purpose of the study, and asked them whether they were willing to undergo screening. She screened the interested patients until the target number of 80 patients was reached. All patients were reassured of the voluntary nature of participation, and online informed consent was obtained from them. Afterwards, online questionnaires asking about depressive symptoms, hopelessness, and suicidal ideation were sent to the patients via WhatsApp. The patients who did not return the questionnaires were contacted by telephone and encouraged to complete and return the questionnaires. Only one follow-up attempt was made for each patient. The data were collected anonymously, without name lists. They were reassessed after 2 months and 5 months using the online questionnaires, and the post- and follow-up scores were obtained (Fig. 1).

### Measures

Data collection in this study was conducted using online forms and questionnaires. Sociodemographic characteristics such as age, gender, marital status, and level of education, as well as clinical features including the duration of depressive symptoms and a history of suicide attempts and psychiatric admission, were evaluated using sociodemographic and clinical assessment forms developed by the research team. The outcome measures used in the study were as follows:

### Primary clinical outcome

The primary clinical outcome in this study was hopelessness, as it has been reported that hopelessness can predict the onset of a major depressive episode^47^.

Patients' hopelessness was evaluated by the Persian version of the Beck Hopelessness Scale (BHS). The BHS consists of 20 self-reported true–false statements that measure the degree of pessimism and negativity about the future. The total scores on this scale range from 0 to 20, and high scores indicate greater hopelessness. Scores 0–3 indicate minimal hopelessness; scores 4–8 indicate mild hopelessness; scores 9–14 indicate moderate hopelessness; and scores 15–20 indicate severe hopelessness^48^. The reliability of this scale was reported by Beck to be 0.69 after one week and 0.66 after six weeks^49^. In the study of Dejkam et al. in Iran, the correlation of the scores of each question with the total score showed that the Beck hopelessness scale measures a single construct, and the reliability of the test was obtained using Cronbach's alpha of 0.79^50^. In the study of Goudarzi et al., the reliability of the Persian version of this questionnaire was reported as 79.0 by Cronbach’s alpha method. Also, the construct validity of this questionnaire was confirmed using the correlation evaluation test of this scale with the Beck depression questionnaire (r = 0.70)^51^. This questionnaire has acceptable internal consistency with a Cronbach’s alpha of 0.79 in the current sample.

### Secondary clinical outcome

Depressive symptoms were assessed using the Beck Depression Inventory Short Form (BDI-SF), which is a valid tool for identifying moderate and severe depression^52^.

This questionnaire has 13 items on a 4-point Likert scale, which are graded from 0 to 3. The maximum score in this questionnaire test is 39, and the minimum score is zero. It has been shown that those who have scores higher than 10 most likely have moderate to severe depression^53^. The Persian version of this scale is a reliable instrument (Cronbach’s alpha = 0.89) for depression. The construct validity of this questionnaire was confirmed by the correlation of its total score with 21 items of the BDI (r = 0.67)^54^. In the present sample, this questionnaire has adequate internal consistency (Cronbach’s alpha = 0.84).

The Beck Scale for Suicide Ideation (BSSI)^55^ was used to evaluate suicidal ideation. This self-reported 19-item scale includes five screening items to assess the presence and intensity of a patient's thoughts, plans, and intentions to commit suicide. Patients who reported positive scores (scores 1 and 2) on the five screening items were considered to have sufficient ideation to require administration of the complete scale^56^. The items are rated on a 3-point Likert scale (0 to 2), with a total score ranging from 0 to 38. No cut-off point was used to categorize the scores, and increasing scores indicate greater suicide risk^56^. The Persian version of the scale had a Cronbach’s alpha coefficient of 0.829 for the screening part and 0.837 for the entire scale^57^. The construct validity of the scale was evaluated by correlating the BSSI scores with the scores of the Symptoms Checklist-90-Revised scales (SCL-90-R)^57^.

### Feasibility, adherence, and satisfaction with the mobile-based logotherapy

The feasibility was evaluated using the percentage of eligible patients who enrolled in the study and remained until the end. If at least 70% of the patients adhered to the study, we considered it feasible^58^. The number of modules and exercises that patients completed (based on self-report) was defined as adherence to the intervention. Full adherence was defined as completing all weekly modules and providing feedback on weekly exercises.

To assess the degree of satisfaction with the training, the Client Satisfaction Questionnaire adapted to internet-based interventions (CSQ-I) was used^59^. The participants completed this 8-item questionnaire online, with responses rated on a 4-point Likert scale ranging from 1 (does not apply to me) to 4 (does totally apply to me). Thus, the scale’s total score ranged from 8 to 32, and it showed excellent internal consistency (McDonald’s omega = 0.93–0.95) and both convergent and discriminant validity^59^. The Persian adaptation of CSQ-I also demonstrated excellent internal consistency in our previous research (Cronbach’s alpha = 0.92)^60^ and current study (Cronbach’s alpha = 0.94). Additionally, in our previous study, the construct validity of the Persian version of the CSQ-I was established by significant correlations between its score and changes in resilience (r = 0.41) and perceived stress (r = 0.54) scores^60^.

### Sample size

Based on the findings of a previous study (mean difference = 7.09, standard deviations: S1 = 7.62, S2 = 10.65)^61^, using the MedCalc software assuming a two-tailed test, α = 0.05, β = 0.99, and 25% attrition, about 80 participants were needed to detect a significant difference between the intervention and control groups.

### Statistical analysis

We used descriptive statistics such as means, standard deviations (SDs), frequencies, and percentages to assess the demographic characteristics of the sample as well as the feasibility, adherence, and satisfaction of the mobile-based logotherapy program. The normality of the distribution of the dependent variables at the levels of the independent variable was assessed using the Kolmogorov–Smirnov test to verify the assumptions of using parametric tests. Demographic variables were compared between groups using Student’s t-test for independent samples and χ^2^ tests. The independent variable in this study was the treatment group, categorized as the intervention group (receiving mobile-based logotherapy and sertraline) and the control group (sertraline plus education about pharmacotherapy), and the dependent variables were the severity of depression, hopelessness, and suicidal ideation measured at three different time points: pre-treatment (T0), post-treatment (T1), and follow-up (T2). A repeated measures MANOVA was conducted to determine whether the mobile-based logotherapy program resulted in significant improvements in depression, suicidal ideation, and hopelessness in patients with major depression.

Effect sizes were calculated using η2 and Cohen’s d, with Cohen’s classification system for effect sizes used to categorize them as small (d = 0.2), medium (d = 0.5), or large (d = 0.8)^62^. The statistical analysis was performed using IBM SPSS Statistics (version 22, IBM Corp., Armonk, NY, USA). The statistical significance was determined by a P-value < 0.05.

### Qualitative phase

At this stage, the participants in the intervention group were selected using purposive sampling. After providing verbal explanations and obtaining online informed consent from participants for participating in the study and recording their voices, telephone interviews were held while considering the ethical principles of research.

Each interview lasted between 30 and 45 min. Data were collected using semi-structured interviews. Each interview started with open-ended, general questions such as, “How has the WhatsApp educational program affected your behaviors, needs, or feelings?” “What changes did you feel in yourself after receiving these trainings?” and continued with, “How do you think the educational program was able to have this effect on you?” “Could you give an example of an anecdote that relates to this?” After the patient's response, subsequent questions were made to further clarify the matter. In addition, probing questions such as “Can you describe more?” “Could you provide an example so I can better understand you?” and “What do you mean?” were asked, as appropriate to the responses.

Following each interview, transcripts were reviewed multiple times and transcribed immediately. Data analysis was performed using the conventional content analysis method developed by Graneheim and Lundman^63^. The primary stages of content analysis included selecting the unit of analysis, organizing the data using open coding, classifying the data based on similarities and differences, reducing the data, and extracting themes.

To ensure the rigor of the data, Guba and Lincoln’s criteria were utilized^64^. The credibility and confirmability dimensions were addressed through prolonged engagement with the study participants, which lasted approximately 6 months. The initial coding of the data was also reviewed by other members of the research team and two qualitative researchers who were not involved in the study to improve the credibility criterion. To ensure the conformity of the researcher’s interpretations with the participants’ experiences, the qualitative results were assessed by the participants themselves. Additionally, the use of proper interview techniques, evaluation of the qualitative data analysis with the research team, and a peer review process were employed to ensure the confirmability and dependability of the data. To address the transferability dimension, a clear description of the qualitative method and its results was provided.

Following the completion of qualitative data analysis, the quantitative and qualitative data were compared and merged, and the results of both stages were evaluated for homogeneity or heterogeneity. After completing quantitative and qualitative data collection and analysis, the common themes and patterns that emerged from the qualitative data with the numerical results obtained in the quantitative study was compared to assess the efficacy of a mobile-based logotherapy program combined with sertraline in the intervention group. In this line, the evaluation of homogeneity or heterogeneity involved assessing whether the patterns and themes derived from qualitative data converged or diverged with the quantitative findings, ensuring a comprehensive understanding of the study outcomes. In addition, the qualitative phase results were used to provide a more detailed interpretation and description of the quantitative research findings.

### Ethical considerations

The study was conducted in accordance with the human subjects’ protection principles outlined in the Declaration of Helsinki and was approved by the Ethics Committee of the Shiraz University of Medical Sciences (IR.SUMS.REC.1400.238). Additionally, this study was registered in the Iranian Clinical Trials Registry under the number IRCT20210622051664N1 on 24/7/2021. Throughout all phases of the study, research ethics principles such as confidentiality of participants’ information and obtaining informed consent were observed. All participants were informed about the research project and their right to withdraw from it. Following the completion of data collection, educational content was provided to the control group participants via WhatsApp.

### Ethics approval and consent to participate

This study received approval from the Ethics Committee of Shiraz University of Medical Sciences under the reference number IR.SUMS.REC.1400.238. The study was conducted in adherence to the principles of the Declaration of Helsinki, and all participants provided written consent after being informed about the study procedures. The research methods were performed in accordance with the applicable guidelines and regulations.

## Results

### Quantitative phase

Of the 80 eligible patients who expressed their willingness to participate in the study, 70 patients (n_intervention_ = 35, n_control_ = 35) completed it. Ten patients had to be excluded from the study: three patients were excluded because they received other medication, and the other seven patients were excluded due to not completing the questionnaires in the Post-treatment and Follow-up Assessments [see Consolidated Standards of Reporting Trials (CONSORT) diagram, Fig. 1].

The mean age of the participating patients was 34.27 ± 7.34 years. The majority of patients were women (81.43%), married (50%), and had an undergraduate degree (57.14%) (Table 2). The mean duration of depressive symptoms (months) in participants in the experimental and control groups was 1.51 ± 0.91 and 1.67 ± 0.96, respectively. Furthermore, most of the patients did not have a history of psychiatric admission (97.1%) or suicide attempts (81.4%). There were no significant differences between the two groups regarding demographic and clinical characteristics (Table 2). The Kolmogorov–Smirnov test showed the normal distribution of the numerical variables. This test assesses whether the data follows a normal distribution. The results of the Kolmogorov–Smirnov test indicated that the assumption of normal distribution was met for variables at all assessment time points (P > 0.05), justifying the use of parametric statistical tests for subsequent analyses. Detailed information about the result of the Kolmogorov–Smirnov test can be found in the Supplementary Materials (Supplementary 1).

**Table 2:** Sample characteristics (n = 70).

Characteristic	Total N = 70	Control N = 35	Experimental N = 35	Value	P-value
Age, M (SD)	34.27 (7.34)	34.86 (7.49)	33.68 (7.25)	− 0.66 (t)	0.51
Gender, n (%)					
Male	13 (18.57%)	5 (14.26%)	8 (22.86%)	0.850 (χ^2^)	0.54
Female	57 (81.43%)	30 (85.71%)	27 (77.14%)		
Marital status, n (%)					
Married	35 (50%)	17 (48.57%)	18 (51.43%)	3.06 (F)	0.38
Single	29 (41.43%)	15 (42.86%)	14 (40%)		
Divorced/widowed	6 (8.57%)	3 (8.6%)	3 (8.57%)		
Educational level, n (%)					
From Primary education to Diploma	27 (38.57%)	15 (42.86%)	12 (34.29%)	0.77 (F)	0.68
Undergraduate degree	40 (57.14%)	19 (54.28%)	21 (60%)		
Postgraduate degree	3 (4.29%)	1 (2.86%)	2 (5.71%)		
History of suicide attempts, n (%)					
Yes	13 (18.6%)	6 (17.1%)	7 (20%)	0.094 (χ^2^)	1
No	57 (81.4%)	29 (82.9%)	28 (80%)		
History of psychiatric admission, n (%)					
Yes	2 (2.9%)	1 (2.9%)	1 (2.9%)	0.000 (F)	1
No	68 (97.1%)	34 (97.1%)	34 (97.1%)		

*M (SD)* mean (standard deviation), *n (%)* number (percent).

The results of the repeated measures MANOVA indicated a significant multivariate main effect of time (Wilks’ Lambda = 0.007, F(6,63) = 1471.851, ɳ^2^ = 0.993, P < 0.001), indicating a significant change in the set of outcome variables over time. The multivariate main effect of group was also significant (Wilks’ Lambda = 0.563, F(3,66) = 17.062, ɳ^2^ = 0.437, P < 0.001), indicating a significant difference in the set of outcome variables between the two groups. Furthermore, there was a significant interaction effect between group and time (Wilks’ Lambda = 0.294, F(6,63) = 25.218, ɳ^2^ = 0.706, P < 0.001), indicating a significant differential effect of time on the set of outcome variables for the two groups.

The univariate tests showed that there were significant main effects of the group on severity of depression (F[1/68] = 19.639, ɳ^2^ = 0.224, P < 0.001), hopelessness (F[1/68] = 17.687, ɳ^2^ = 0.206, P < 0.001) and suicidal ideation (F[1/68] = 6.886, ɳ^2^ = 0.092, P = 0.011). This means that ignoring the effect of time, there were significant differences among the groups regarding marginal means of severity of depression, hopelessness, and suicidal ideation. There were also significant main effects of time on the severity of depression (F[2/119.31] = 1399.761, ɳ^2^ = 0.954, P < 0.001), hopelessness (F[2/136] = 2216.187, ɳ^2^ = 0.970, P < 0.001) and suicidal ideation (F[2/97.86] = 194.017, ɳ^2^ = 0.740, P < 0.001). This means that ignoring the effect of group, there were significant differences among the times regarding marginal means of severity of depression, hopelessness, and suicidal ideation.

The interaction of group × time for severity of depression, hopelessness and, suicidal ideation was statistically significant (Table 3). This means that the change in severity of depression, hopelessness, and suicidal ideation over time is different depending on group membership. The interaction effect of group × time on the severity of depression (F[2/119.31] = 6.654, η^2^ = 0.089, P = 0.003) indicates that there are statistically significant differences in the change in severity of depression over time between the intervention and control groups. Specifically, the intervention group, which received an 8-week mobile-based logotherapy program in addition to sertraline, experienced a statistically significant reduction in the severity of depression compared to the control group, which received only sertraline and education about pharmacotherapy during the time. The effect size (η^2^ = 0.089) suggests that 8.9% of the variance in depression severity can be attributed to the group × time interaction.

**Table 3:** One-way repeated measures MANOVAs, F-ratios, P values, and partial ɳ^2^.

Measures	F (df)	P value	ɳ^2^
Between subjects			
Group			
Depression (BDI-13)	19.639 (1,68)	< 0.001	0.224
Hopelessness (BHS)	17.687 (1,68)	< 0.001	0.206
Suicidal ideation (BSSI)	6.886 (1,68)	0.011	0.092
Within subjects			
Time			
Depression (BDI-13)	1399.761 (2,119.31)	< 0.001	0.954
Hopelessness (BHS)	2216.187 (2,136)	< 0.001	0.970
Suicidal ideation (BSSI)	194.017 (2,97.86)	< 0.001	0.740
Time × group			
Depression (BDI-13)	6.654 (2,119.31)	0.003	0.089
Hopelessness (BHS)	56.629 (2,136)	< 0.001	0.45
Suicidal ideation (BSSI)	7.720 (2,97.86)	0.002	0.102

*df* degrees of freedom, *BDI-13* Beck Depression Inventory Short Form Items, *BHS* Beck hopelessness scale, *BSSI* Beck Scale for Suicide Ideation.

Similarly, for hopelessness, the interaction effect of group × time (F[2/136] = 56.629, η^2^ = 0.45, P < 0.001) reveals substantial differences in the change in hopelessness over time between the two groups. In the intervention group, the combination of mobile-based logotherapy and sertraline led to a significant reduction in hopelessness compared to the control group. The effect size (η^2^ = 0.45) indicates that 45% of the variance in hopelessness can be attributed to the group × time interaction.

Regarding suicidal ideation, the interaction effect of group × time (F[2/97.86] = 7.720, η^2^ = 0.102, P = 0.002) signifies meaningful differences in the change in suicidal ideation over time based on group membership. The intervention group experienced a statistically significant decrease in suicidal ideation compared to the control group. The effect size (η^2^ = 0.102) suggests that 10.2% of the variance in suicidal ideation can be attributed to the group × time interaction.

Multiple pairwise comparison tests (Bonferroni) between pre-treatment and post-treatment (T0-T1) and between pre-treatment and follow-up (T0-T2) showed that both groups had a significant improvement in the severity of depression, hopelessness, and suicidal ideation at post-treatment and follow-up. Means, SDs, and Cohen’s d effect sizes for dependent variables at the different measurement times are presented in Table 4.

**Table 4:** Repeated measures MANOVAs, means (SDs), and effect sizes (Cohen’s d).

Measure		Pretreatment M (SD)	Posttreatment M (SD)	Follow-up M (SD)	Pre–post effect size, d	Pre-Follow up effect size, d	Bonferroni tests Mean difference	
							Pre–post	Pre-follow up
Depression	Control	34.98 (1.92)	22.14 (2.59)	11.54 (3.21)	5.63	8.86	12.84***	23.44***
	Experimental	35.26 (2.03)	20.32 (2.47)	8.37 (3.74)	6.61	8.94	14.94***	26.89***
Hopelessness	Control	19.77 (1.86)	8.31 (1.18)	5.00 (1.28)	7.36	9.25	11.46***	14.77***
	Experimental	22.00 (2.18)	6.20 (2.04)	1.60 (1.56)	7.48	10.76	15.80***	20.40***
Suicidal ideation	Control	16.97 (6.81)	9.31 (5.19)	4.80 (1.28)	1.26	2.48	7.66***	12.17***
	Experimental	18.34 (8.55)	5.77 (2.07)	0.60 (0.65)	2.02	2.92	12.57***	17.74***

*M (SD)* mean (standard deviation).

***P < 0.001.

**Figure 2:**
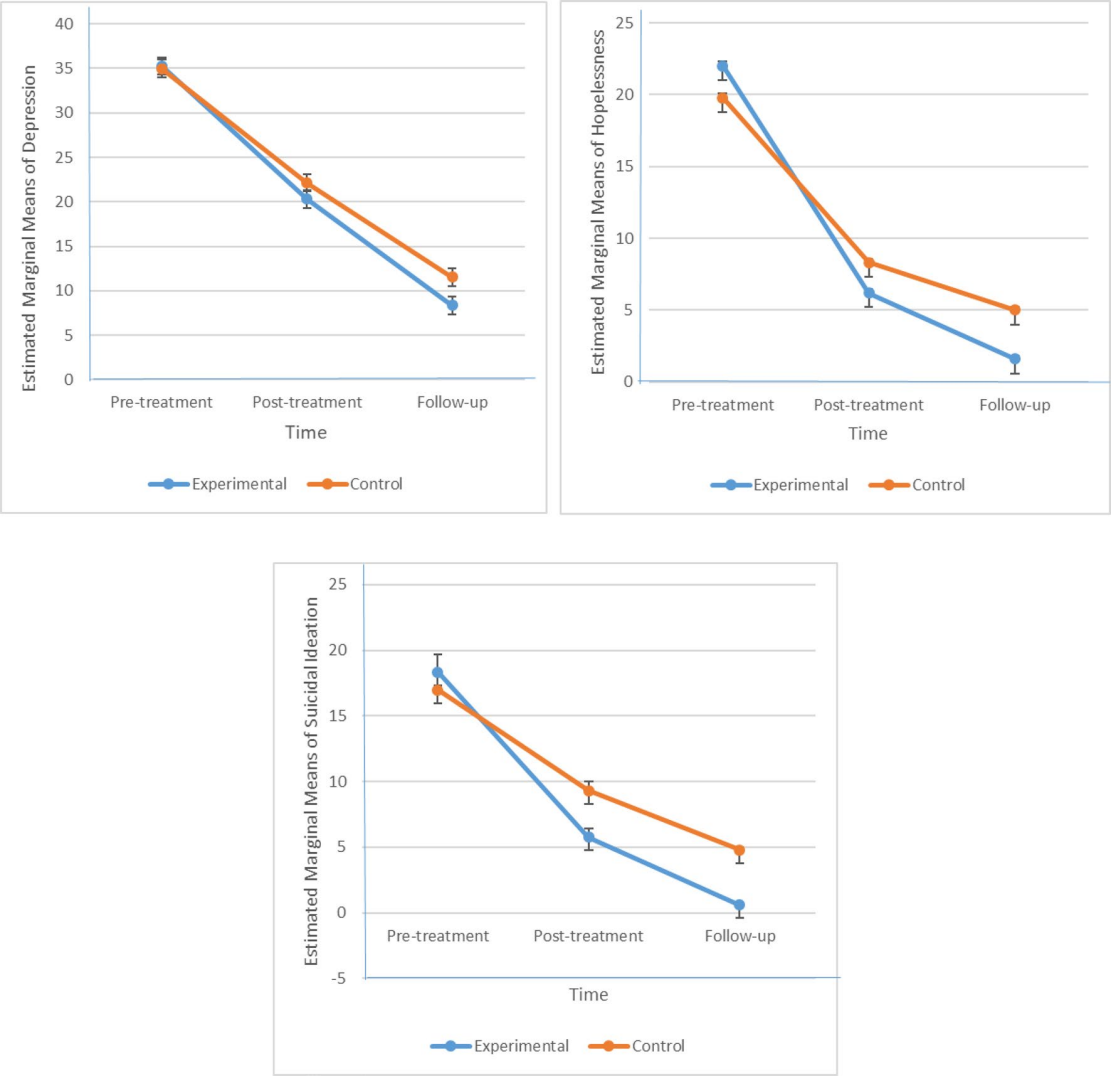
Estimated changes in depression, hopelessness, and suicidal ideation from baseline to 3-month follow-up.

The means of the severity of depression, hopelessness, and suicidal ideation in experimental and control groups are depicted in Fig. 2.

Out of the 40 patients who received both logotherapy and pharmacotherapy, 35 (87.5%) completed the post-assessments at T2. Three patients were excluded from the study due to receiving other medications, and two were excluded due to not completing the questionnaires. Among the 35 patients who completed the post-assessments, 27 (77.14%) adhered fully to the interventions by completing all 8 modules and providing feedback on the exercises. The remaining patients did not fully adhere to the interventions, with four completing all eight modules but not providing feedback, two completing 75% of the modules, and two completing 50% of the modules. In the control group, all 40 patients started the study, and 35 (87.5%) completed the post-assessments at T2. The remaining five patients were excluded from the study due to not completing the post-test questionnaires.

Participants in the intervention group reported high levels of satisfaction with the mobile-based education program. Mean scores on the satisfaction items ranged from 3.42 (SD = 0.5) on item 3 (This education has met my needs) to 4 (SD = 0) on items 4 (If anyone needs similar help, I recommend this training), 7 (In general, I am satisfied with the training), and 8 (I would come back to such a training if I were to seek help again). The average total score on the CSQ-I was 30.05 (SD = 0.99), indicating high satisfaction with the program among participants in the intervention group.

### Qualitative phase

A total of 12 participants (8 females and 4 males) from the intervention group were invited to participate in semi-structured, in-depth phone interviews. The participants’ ages ranged from 31 to 46 years. Table 5 presents the demographic characteristics of these participants. The purpose of the interviews was to provide further explanation of the findings from the quantitative stage. Twelve telephone interviews were conducted in total.

**Table 5:** Demographic characteristics of the participants.

No	Age	Gender	Education	Job
P1	40	Female	BSc	Home keeper
P2	41	Female	BSc	Employee
P3	36	Female	MSc	Employee
P4	31	Female	BSc	Home keeper
P5	41	Female	High school	Self-employed
P6	38	Female	BSc	Employee
P7	34	Male	High school	Self-employed
P8	40	Male	BSc	Self-employed
P9	35	Female	BSc	Employee
P10	42	Female	BSc	Home keeper
P11	33	Male	High school	Self-employed
P12	46	Male	BSc	Employee

Data analysis resulted in three main categories: “efficient instruction”, “user-friendly intervention” and “constructive change”. Table 6 presents the categories and subcategories obtained from the interviews.

**Table 6:** Main themes, categories, and sub-categories in the qualitative stage.

Themes	Categories	Sub-categories
Efficient instruction	Useful educational content	Informative educational content
		Quality educational content
	General usability of instructions	Relatives and acquaintances usage from educational program
		Applicability for any age group and level of education
	Frequent usage	Returning to education during the study
		Continuing use after the study
User-friendly intervention	Interesting educational content	Attractive educational messages
		Tendency to train
	Convenient use	Access to training at any condition
		Easy training
		Comprehensible educational content
		Non-time-consuming content
		Free access to education
	Respect for the client’s privacy	Client anonymity
		Training in private
Constructive change	Positive cognitive changes	Getting rid of suicidal thoughts
		Finding meaning
		Increasing awareness
		Creating positive thoughts
		Creating realistic expectations
	Emotional state improvement	Promoting relaxation in the client
		Creating positive feelings and energy
		Inducing hope
		Enjoying life
		Improve the feeling of happiness
		Transition from the emotional crisis (feelings of disappointment, anger control, reducing tension, and stress)
	Interpersonal relationship improvement	Family relationship improvement
		Social relationship improvement
	Building good habits	Live in the moment
		Dealing with interests
		Create positive changes in the daily routine

#### Efficient instruction

Three categories were identified for the “efficient instruction” theme, namely “useful educational content”, “general usability of instructions” and “frequent usage”. The participants believed that the WhatsApp educational program was useful and informative for them, and they have used the educational materials many times. They also found the educational contents useful for other healthy family members and relatives.

##### Useful educational content

The participants described the educational contents as high quality and informative, which helped them face challenges and feel relaxed and positive. A participant stated:

- I was satisfied with the educations. It was useful for me. I mean, I felt that it was something instructive for me to know how to enjoy, how to keep your peace, how to control your violence, how to be thankful, how to use what I have, and what kind of blessing it is. Very good and effective content. (P2)

One of the participants stated that the training, besides medicine and therapy, produced a positive effect on her. Although she believed that the effect of training is much better since it has given her a better motivation to live than medicine therapy.

- The training had a good effect on me. I can’t say that it was just because of the medicine; I think the training was much better. That is, I feel that training gave me a better motivation to live than medicine. (P6)

##### General usability of instructions

The participants recommended that not only themselves but also their family and relatives use the educations. They believed that the educational materials were practical and effective in improving their mental health.

- The education was really good for me. Considering that my daughter is a teenager, many of the group’s contents are really good for her. That’s why I used to send him a lot of his educational material, so that it would have an effect on him. Myself and my daughter tried to practice a lot of these educational materials. I used to tell my daughter to write down the key things, keep them for yourself, and do them. (P10)

The participants in the intervention group believed that the educational program was feasible for people of any age and level of education.

- They were useful educations for people of any age; for example, myself, my mother, and my son, who is a student, used a lot of educations. The educations were comprehensible and applicable to every age group. (P12)

##### Frequent usage

The participants in the intervention group used the educational materials frequently and in various times, places, and situations, including mental difficulties and distress.

- I used to take notes; it was very good. I still use it when bad thoughts come to me and when I get frustrated. (P4)
- You could use its’ exercises and educations in any situation, day or night. You could watch these clips again and use these exercises at other times. I think it is very good. (P1)

### User-friendly intervention

The participants in the intervention group clearly experienced the educational interventions as user-friendly. Categories associated with this main theme were “interesting educational content”, “convenient use” and “respect for the client’s privacy”. The participants were interested in the educational program. The participants found the intervention accessible, easy to use, and not time consuming. They received their education privately and anonymously.

#### Interesting educational content

The participants tended to use the educational materials because they found the educational messages and contents so interesting and attractive. Moreover, the use of new technology and different educational methods (clips, exercises, etc.) affected the attractiveness of the educational materials.

- The content was completely good, informative, brief, understandable, and applicable. The educational content was not only in written form, which you don’t tend to go to; it also had practical and short exercises, and we could have various files through WhatsApp, a public and available app. That’s why I always watched with interest in the group when there was a new message in the group. I was eager to read what the program is today, what he wants to talk about, and what he wants to teach. (P8)

#### Convenient use

The participants experienced the convenience of using the educational interventions through WhatsApp. They could access the easy and comprehensible educational contents at any time and for free. The participants mentioned that educational contents provide key beneficial strategies that do not take much time from them.

- The strategies and contents were very simple but had a high positive feeling, and they were free. That’s why I like them so much. It could be touched and implemented in real life. (P7)
- Because when you want to go to a clinic, hospital, or… a place to learn these things, it takes time, and you may not have enough time to do it, but in this case, it's much better to be in your room and have your cell phone next to you, by which you learn. (P7)

#### Respect for the client’s privacy

The participants received the educations not in person. They preferred to remain anonymous to the other members of the group while receiving training.

- No one knows anyone when they teach us, you know! This was good for me. The private state of the person is preserved. (P11)

However, at some times and in psychological crises, the participants believed that using the face-to-face educational method along with these virtual educations could be more beneficial for the patient.

- In my opinion, for those who have reached the extreme state of their illness, talking and having a face-to-face conversation are much more effective than studying texts or watching a movie in a virtual group. (p3)

#### Constructive change

Main themes associated with constructive change were “positive cognitive changes”, “emotional state improvement”, “interpersonal relationship improvement” and “building good habits”. The participants experienced an improvement in their emotional and cognitive situations and interpersonal relationships. The participants believed that the educational program helped them create positive and interesting habits in their lives.

##### Positive cognitive changes

The WhatsApp educational program had significant effects on the cognition of participants in the study. In the view of the participants, the education improved their awareness of effective strategies to face their challenges and depressive episodes. The participants believed that utilizing the educational program caused them to think positively; moreover, they could find a new pleasant meaning for life following the education. The participants believed that the educational program had modified their unrealistic expectations.

- Life has a better meaning with these techniques, and I feel much better. (P3)
- My life was a very good life, but I didn't see it; I was thinking a lot about dying. In my opinion, the educations made me more realistic and see my life much better. Giving thanks helped me get out of suicidal thoughts. (P6)

##### Emotional state improvement

The participants experienced peace and happiness after using the educations. They believed that the educational contents produced positive feelings, energy, and hope in them.

- The educational program made me feel hopeful and gave me a positive feeling. (p5)
- When you do the recommended exercises in the WhatsApp group, you feel good, you feel positive, and your energy increases. (P2)

##### Interpersonal relationship improvement

The interpersonal relationships improved after getting the educational program via WhatsApp.

- I reduced my relationship with my family. For example, even though we were very close, we didn’t see each other for two weeks. But I’m not like this anymore. I am more satisfied with the presence of others in my life. (P6)
- In my family, I used to have a cold relationship with my husband, but now I don’t have this feeling; I'm back to a normal life, and it’s very good. (P6)

##### Building good habits

Building good habits was another category of the theme of “constructive change”. After receiving education, the participants developed the useful habit of living in the moment. They also allocated their leisure time to dealing with their own interests. The participants also changed some of their ordinary and usual habits (such as sleep patterns) in a positive and effective way after receiving the education.

- There were some habits that were changed after the education. For example, it made me wake up earlier in the morning on days when I didn’t want to go to work. (P6)
- I was not like this before. For example, I was very interested in flowers and plants, but I felt that I couldn’t handle them. But now I have a very small greenhouse, and I take care of the flowers when I am at home. It has had a very good effect on me. I spend all my time at home usefully. (P6)
- Well, look, the education made me understand that I should not dwell on the past and regret the past, and the future is not as important as the present. (P3)

## Discussion

The present study utilized both qualitative and quantitative methods to investigate the effect of mobile-based logotherapy via WhatsApp on the symptoms of depression, suicidal ideations, and hopelessness in patients with major depression. The results of the quantitative phase of this study showed both sertraline combined with mobile-based logotherapy and also sertraline combined with education about pharmacotherapy significantly decreased the symptoms of depression, suicidal ideations, and hopelessness in patients with major depressive disorder, but the positive effect of sertraline combined with mobile-based logotherapy on depression, suicidal ideations, and hopelessness in the intervention group was significantly greater than another intervention performed for the control group during the time. The qualitative research conducted on the intervention group confirmed the efficacy of sertraline plus mobile-based logotherapy through WhatsApp in the intervention group and provided valuable insights into the factors that may have influenced this effectiveness.

The findings of this study are consistent with previous research that has demonstrated the effectiveness of combining pharmacotherapy with psychotherapy or other non-pharmacological interventions for the treatment of major depression^17,65,66^. According to Shaygan et al.’s research, relying solely on pharmacotherapy may not be adequate for preventing suicidal ideation in patients with major depressive disorder, particularly during the initial stages of sertraline treatment. Instead, a combination of pharmacotherapy and psychotherapy may offer a more effective approach for treating major depressive disorder over both the short and long term^17^. Logotherapy is a type of psychotherapy that focuses on assisting individuals in discovering meaning and purpose in their lives, even when facing challenging situations^20^. Integrating logotherapy into the treatment regimen of major depressive disorder may enable the biological and psychological components of the illness to be addressed, resulting in a more comprehensive approach to treatment. Based on the findings of the qualitative phase of this study, the logotherapy program via WhatsApp, besides using antidepressant drugs, could have a positive effect on the depression symptom. However, the effect of training is much better than medicine therapy since it gives the patient better insight and motivation about life. It suggests that a logotherapy program can have significant psychological effects on patients with MMD that cannot be ignored in the treatment process for these patients.

The results of many studies have shown the efficacy of logotherapy in decreasing depression symptoms and suicidal ideation and improving hope in different groups of people (such as physically disabled women, older adults, patients with leukemia and spinal cord injury, imprisoned women, and women with AIDS)^27,67–72^. For example, the results of the semi-experimental study of Golshan et al. showed that group training through logotherapy (8 sessions of logotherapy, twice a week, each lasting 120 min) significantly decreased depression (P ≤ 0.01) in physically disabled women, and the sustainability of this effect at one month follow-up^67^. In another study, Ghelbash et al. conducted a clinical trial study with a pre-test and post-test design. In this study, the experimental group received 10 sessions of group logotherapy (two group sessions per week, each lasting 90 min), but the control group received only general interventions provided by the prison, which included attending vocational training classes and individual counselling on several occasions. Based on this study, group logotherapy had a significant effect on increasing imprisoned women’s level of hope^71^. In another instance, Kamae et al. conducted a semi-empirical study using the pre–post-test design with a control group. In this study, the intervention group received 10 logotherapy sessions once a week, held for 2 h, but during the treatment period, the control group did not receive any psychological services. The results of this study revealed that providing logotherapy can reduce suicidal thoughts in women with AIDS^72^.

Based on a few studies conducted on people with depression^29,31,33,34^, logotherapy could be effective in reducing the level of depression^29,33,34^, hopelessness^29,31^ and suicidal ideation in this group of patients^29^. For instance, Sun et al. conducted a quasi-experimental study on patients with major depressive disorder in Taiwan. In this study, the experimental group received 4–6 logotherapy interviews in 12 weeks, and the control group only received depression education as usual. Based on the results of this study, logotherapy was an effective method for increasing meaning in life and reducing the degree of depression, hopelessness, and suicidal ideation in patients with depression^29^. It is worth mentioning that although this study was so similar to the current study in some aspects, there are significant differences between this study and the current study, including a quasi-experimental study design, the legotherapy method (individual face-to-face legotherapy), different interventions (logotherapy interviews in the experimental group vs. depression education in the control group), and different intervention session numbers and duration. In addition, despite the current study, the logotherapy educational content was based on the participants’ attitudes about the meaning of life that were mentioned during the interview. In another study, Esalati et al. performed a quasi-experimental study on students with depression using a pre-test and post-test design with a control group. In this study, the experimental group was trained for 8 sessions of 90 min (twice a week) using Frankl’s logotherapy method, while the control group was placed on the waiting list for training. The intervention was implemented with the methods of lectures, questions and answers, discussion, and assignment presentation in groups. The results indicated that group logotherapy increased psychological well-being, which improved the condition of depression in students with depression^34^. Similar to the present study, in this study the various aspects of logotherapy, including frankl logotherapy approach, finding the meaning of life and also the meaning of suffering and painful events, accepting depression as a meaningful event, training about the meaning source, providing strategies to make positive meaning, etc., were covered in the educations. However, despite the present study, some important logotherapy issues in making meaning of life, such as nature, death, thanksgiving, etc., were not addressed in the logotherapy sessions. Mohammadi et al. carried out a quasi-experimental study with a pre-test-post-test design with a control group of women suffering from depression. In this study, the experimental group underwent ten 90-min weekly sessions of logotherapy, while the control group didn’t receive any intervention. The results of this study showed that logotherapy has been effective in increasing hope for life in women suffering from depression^31^. However, in this study, no explanation has been provided regarding the content of logotherapy sessions. Hamid and Wasy conducted a semi-experimental study on women with neurotic depression that included a pre-test and post-test, a follow-up period, and a control group to investigate the effect of face-to-face group logotherapy. In this study, the experimental group received 12 sessions of face-to-face group logotherapy combined with the recitation of verses from the Holy Quran and prayer, but the control group did not receive any intervention. The results revealed that there was a significant difference between the experimental and control groups in terms of depression symptoms. These results continued significantly during the follow-up period^33^. It is worth mentioning that a significant part of the contents of the logotherapy were related to religious subjects, such as the recitation of verses from the Holy Quran and psychotherapy prayers.

Regarding the assessment of study design, it has to be considered that, despite the current RCT study, a quasi-experimental design was used in all of these studies. The benefit of a well-designed clinical trial compared to a quasi-experimental study lies in its capacity to establish causation and offer more robust evidence for the efficacy of a treatment. A clinical trial provides greater control over confounding variables and can generate high-quality evidence that informs clinical decision-making and the development of guidelines^73^. On the other hand, while Major depressive disorder (MDD) is among the most common mental disorders worldwide, generating great functional impairment and very high economic expenditure for the health systems^74,75^, most of the previous studies assessed the effect of logotherapy on people with other types of depression than major depression, and the participants’ use of antidepressant medication wasn’t considered^31,33,34^. Therefore, in this study, these important matters were covered. The use of sertraline is consistent with current treatment guidelines for major depression, which recommend the use of antidepressant medication as a first-line treatment option^76^. So, the use of sertraline in both the intervention and control groups in the present study allowed for a more controlled comparison between the two interventions, as both groups were receiving the same medication treatment. This can help to rule out any potential confounding effects of medication on the study results and provide a clearer understanding of the specific effects of the interventions being studied.

The method used to provide logotherapy to the participants in all related studies was face-to-face (individual or group). It seems using the virtual method of group therapy may be considered another strong point of this study. One advantage of group therapy is that it is less time-consuming than individual therapy^77^. Also, group therapy provides opportunities for group members to acquire new social skills and behaviors, improve social relationships, and learn from the common experiences of other members of the group. These factors can lead to improvements in the treatment and improvement of psychological problems caused by individuals’ diseases^78^. Nonetheless, some people may not be a good fit for or may not be used to attending face-to-face group therapy due to a lack of personal safety or not liking to talk in public^77^. In this line, the participants in the qualitative phase of this study prefer to use educational logotherapy and share personal issues in group without revealing their personal identity to other group members. A good solution for this issue is virtual group therapy. Several studies have shown promising results of smartphone-based mental health interventions^79–81^ and social media such as WhatsApp^82,83^ in reducing symptoms of depression and improving mental health outcomes. However, the results of Linardon et al.’s study showed that Smartphone interventions did not differ significantly from active interventions (face-to-face, computerized treatment)^80,84^. However, the main approaches used in these studies were CBT therapy, mindfulness training, or mood monitoring, which is different from the mobile-based logotherapy intervention used in this study^79,84^. Moreover, these studies have not specifically focused on the effectiveness of interventions using social media, especially WhatsApp, in reducing symptoms of depression and improving mental health outcomes. Studies have suggested that the use of social media, including WhatsApp, for mental health support can lead to increased social support^82,85^, improved self-esteem^86^, reduced symptoms of depression and anxiety^87^, and increased access to mental health resources^82^, making them promising tools for promoting mental health and wellbeing. Additionally, studies have found that individuals use social media to discuss mental health issues for a variety of reasons, including seeking support and information, reducing stigma, and expressing themselves^88^. So it seems that considering social media platforms to deliver the mental health intervention in this study was beneficial. Based on the qualitative results of this study, the use of WhatsApp for providing logotherapy offers several advantages, including increased accessibility, convenience, privacy for patients, cost-effectiveness, and the ability to tailor interventions to individual patient needs. Nevertheless, one participant mentioned that although logotherapy by WhatsApp has been effective and desirable for her, the face-to-face method can be more effective than mobile-based logotherapy for patients when they have severe symptoms of depression. Based on a meta-analysis of randomized controlled trials carried out by Firth et al., With regards to population type, significant benefits of smartphone apps were only found for those with self-reported mild-to-moderate depression^89^. This may be due to variations in subgroup sample sizes, as the majority of studies were conducted in non-clinical populations, thus leaving the analyses for major depression underpowered to detect significant effects. Moreover, the nature and content of smartphone interventions may have an impact on the results of these studies. In this study, it should be considered that the quantitative results of the current study showed the efficacy of mobile-based logotherapy on patients with moderate and severe depression, and other patients who participated in the qualitative phase of this study (some of whom had severe depression) reported the positive effects of mobile-based logotherapy. One of the probabilities for the efficacy of mobile-based logotherapy for people with moderate and severe depression may be that it presents a comprehensive educational logotherapy program suitable for addressing the needs of patients with different severity levels of depression and with an interesting method. Nonetheless, it seems there is a need to conduct further studies to assess the effect of logotherapy on patients with severe MDD.

Moreover, based on the existing evidence, it seems that there were differences in the educational contents of logotherapy sessions in these studies compared to the current study. In this study, the educational intervention of logotherapy covered wider and more diverse areas than other similar studies. Based on the qualitative results of this study, participants reported that the educational contents of logotherapy were interesting, high-quality, and informative, and they used them frequently. They also found the educational materials practical and effective in improving their mental health and that of their relatives, and they recommended them to their family and relatives. The program helped participants develop positive habits and improve their emotional state, cognitive processing, and interpersonal relationships.

Overall, in this study, mobile-based logotherapy via WhatsApp may help patients with major depressive disorder identify their own sense of meaning and purpose, which could contribute to their overall mental health and wellbeing. This is consistent with Frankl’s theory of logotherapy. In this line, meaning in life consists of three dimensions: (1) The cognitive component is coherence, which means a sense of comprehensibility and one’s life making sense; (2) The motivational component is related to setting vital goals and purposes and representing the prospective dimension of meaning in life; (3) The affective component is a feeling of satisfaction and fulfillment in an individual’s life^90,91^. Considering the qualitative results of the current study. It seems that, in this study, logotherapy can create positive changes in the emotional, cognitive, and motivational aspects of patients with major depression. Regarding the design of the intervention based on the three pathways of meaning therapy in the Frankl theory^20^, these results seem reasonable.

Research has demonstrated that the function of meaning in life can serve as a resource for resilience^92^. When individuals find meaning in difficult situations, they emerge stronger, safer, and happier, leading to improved quality of life and greater resilience^93^. Creating moments of meaning in life has also been shown to reduce feelings of despair^94^ and suicide ideation^95^, while meaning in life is positively correlated with optimism and negatively predicts suicidal ideation^96^. Therefore, focusing on meaning and purpose in logotherapy may be particularly beneficial for individuals who are experiencing feelings of hopelessness, as it provides a way to reframe their experiences and find new sources of meaning and purpose in life. The present study suggests that these positive changes may lead to a reduction in symptoms of depression, hopelessness, and suicidal ideation.

### Limitations

Limitations of this study included the relatively small sample size and the use of self-reported measures to assess treatment outcomes. Future research with larger samples and more objective measures of treatment outcomes would be beneficial for further assessing the effectiveness of mobile-based logotherapy combined with sertraline for patients with major depressive disorder. Another limitation of this study is that some patients didn’t have the time or availability to use all of the educational contents of logotherapy or participate in group discussions at the scheduled time; however, frequent contact—through text messages and/or phone calls—with the patients helped to increase the participants’ adherence to the intervention process. The unequal duration of the intervention in the control and intervention groups may be a confounding factor affecting the study's internal validity. However, this design was based on the distinct goals and nature of interventions and enhances the external validity of our study by simulating real-world scenarios where interventions may vary in duration and complexity. Furthermore, although this study determined the efficacy of mobile-based logotherapy as an adjunct to pharmacotherapy, In future studies, it is suggested to compare the effectiveness of mobile-based logotherapy and traditional in-person psychotherapy for treating major depressive disorder. This could explore treatment outcomes, patient preferences, and the cost-effectiveness of psychotherapy methods, offering insights into tailored mental health care.

## Conclusion

In conclusion, the findings of this study suggest that mobile-based logotherapy combined with sertraline through WhatsApp may be an effective treatment option for decreasing symptoms of major depression, hopelessness, and suicidal ideation. Based on the qualitative results of this study, participants in the intervention group considered mobile-based logotherapy through WhatsApp as a user-friendly intervention with efficient instruction that made constructive change in them. It seems that by combining pharmacotherapy with non-pharmacological interventions, such as logotherapy, it is possible to address both the biological and psychological aspects of depression and provide a more comprehensive treatment approach.

Mobile-based logotherapy may be a viable and effective option for individuals who tend to use mobile-based psychotherapy or are unable or prefer not to undergo traditional forms of therapy. In this regard, providing facilities to develop qualified educational content, establishing infrastructure in the area of the mental health care system to deliver mobile-based logotherapy to patients with MDD, and encouraging patients to follow these programs can be beneficial strategies that should be performed by mental health care authorities.

### Supplementary Information

Supplementary Information.

## Data Availability

The datasets generated and/or analyzed during the current study are not publicly available due to patient confidentiality but are available from the corresponding author on reasonable request.

## References

[1] Bhatta DK (2021). Burden of depressive and anxiety disorders in Nepal, 1990–2017: An analysis of Global Burden of Disease Data. MedRxiv.

[2] Johns G, Samuel V, Freemantle L, Lewis J, Waddington L (2022). The global prevalence of depression and anxiety among doctors during the covid-19 pandemic: Systematic review and meta-analysis. J. Affect. Disord..

[3] Deng J (2021). The prevalence of depression, anxiety, and sleep disturbances in COVID-19 patients: A meta-analysis. Ann. N. Y. Acad. Sci..

[4] Pilevarzadeh M (2019). Global prevalence of depression among breast cancer patients: A systematic review and meta-analysis. Breast Cancer Res. Treat..

[5] Li H, Ge S, Greene B, Dunbar-Jacob J (2019). Depression in the context of chronic diseases in the United States and China. Int. J. Nurs. Sci..

[6] Silverman AL, Herzog AA, Silverman DI (2019). Hearts and minds: Stress, anxiety, and depression: Unsung risk factors for cardiovascular disease. Cardiol. Rev..

[7] Aw PY (2023). Co-prevalence and incidence of myocardial infarction and/or stroke in patients with depression and/or anxiety: A systematic review and meta-analysis. J. Psychosom. Res..

[8] Sadock, B. *Kaplan & Sadock’s Synopsis of Psychiatry: Behavioral Sciences/Clinical Psychiatry 76 DJ Herzog* (2020).

[9] Abdi S, Spann A, Borilovic J, de Witte L, Hawley M (2019). Understanding the care and support needs of older people: A scoping review and categorisation using the WHO international classification of functioning, disability and health framework (ICF). BMC Geriatr..

[10] Pompili M (2019). Critical appraisal of major depression with suicidal ideation. Ann. Gen. Psychiatry.

[11] Avasthi A, Grover S (2018). Clinical practice guidelines for management of depression in elderly. Indian J. Psychiatry.

[12] Abramson, L., Metalsky, G. & Alloy, L. The hopelessness theory of depression: A metatheoretical analysis with implications for psychopathology research. *Manuscript Submitted for Publication* (1986).

[13] Troister T, D'Agata MT, Holden RR (2015). Suicide risk screening: Comparing the Beck depression inventory-II, beck hopelessness scale, and psychache scale in undergraduates. Psychol. Assess..

[14] Nekanda-Trepka C, Bishop S, Blackburn IM (1983). Hopelessness and depression. Br. J. Clin. Psychol..

[15] Hallensleben N (2019). Predicting suicidal ideation by interpersonal variables, hopelessness and depression in real-time. An ecological momentary assessment study in psychiatric inpatients with depression. Eur. Psychiatry.

[16] Cipriani A (2009). Comparative efficacy and acceptability of 12 new-generation antidepressants: A multiple-treatments meta-analysis. The Lancet.

[17] Shaygan M, Sheybani Negad S, Motazedian S (2022). The effect of combined sertraline and positive psychotherapy on hopelessness and suicidal ideation among patients with major depressive disorder: A randomized controlled trial. J. Positive Psychol..

[18] Guidi J, Fava GA (2021). Sequential combination of pharmacotherapy and psychotherapy in major depressive disorder: A systematic review and meta-analysis. JAMA Psychiatry.

[19] Cooper M (2016). Existential Therapies.

[20] Frankl VE, Boyne J (2017). Man's Search for Meaning: Young Adult Edition.

[21] Putri SB, Jannah M (2019). The effect of logotherapy on depression in breast cancer patients under chemotherapy. Breast Cancer.

[22] Hamid N, Talebian L, Mehrabizadeh Honarmand M, Yavari A (2011). The effects of logotherapy on depression, anxiety and quality of life of cancer patients in ahvaz big oil haspital. Psychol. Achiev..

[23] Siadat M, Gholami Z (2018). The effectiveness of group logotherapy in increasing resilience and decreasing depression among individuals affected by substance abuse in Tehran. Int. J. Appl. Behav. Sci..

[24] Bahar A, Shahriary M, Fazlali M (2021). Effectiveness of logotherapy on death anxiety, hope, depression, and proper use of glucose control drugs in diabetic patients with depression. Int. J. Prev. Med..

[25] Robatmili S (2015). The effect of group logotherapy on meaning in life and depression levels of Iranian students. Int. J. Adv. Couns..

[26] Kang K-A, Kim S-J, Song M-K, Kim M-J (2013). Effects of logotherapy on life respect, meaning of life, and depression of older school-age children. J. Korean Acad. Nurs..

[27] Shariat A, Yarmohammadian A, Solati K, Chorami M (2021). The effectiveness of logotherapy on depression and positive psychological characteristics of the elderly. Aging Psychol..

[28] Koulaee AJ, Khenarinezhad F, Abutalebi M, Bagheri-Nesami M (2018). The effect of logotherapy on depression in cancer patients: A systematic review study. World Cancer Res. J..

[29] Sun FK (2022). The effects of logotherapy on meaning in life, depression, hopelessness, and suicidal ideation, in patients with depression: An intervention study. Perspect. Psychiatr. Care.

[30] Kim C, Choi H (2021). The efficacy of group logotherapy on community-dwelling older adults with depressive symptoms: A mixed methods study. Perspect. Psychiatr. Care.

[31] Mohammadi F, Fard FD, Heidari H (2014). Effectiveness of logo therapy in hope of life in the women depression. Procedia Soc. Behav. Sci..

[32] Ebrahimi N, Bahari F, Zare-Bahramabadi M (2014). The effectiveness of group logo therapy on the hope among the leukemic patients. Iran. J. Cancer Prev..

[33] Hamid, N. & Wasy, S. *Effectiveness of Logotherapy Together with Quran Recitation and Prayers on Treatment of Depression and t Helper Cell (CD4+)* (2013).

[34] Esalati P, Arab A, Mehdinezhad V (2019). Effectiveness of Frankl’s logotherapy on health (decreasing addiction potential and increasing psychological well-being) of students with depression. Iran. J. Health Educ. Health Promot..

[35] Ebrahimi NBF, Zare-Bahramabadi M (2014). The effectiveness of group logo therapy on the hope among the leukemic patients. Iran. J. Cancer Prev..

[36] Mihandoust SRM, Soleymani M (2021). Logotherapy to improve parent-child relationship among mothers of autistic children: A randomized clinical trial. Eur. Rev. Med. Pharmacol. Sci..

[37] Soetrisno SS, Ardhianto A, Hadi S (2017). The effect of logotherapy on the expressions of cortisol, HSP70, Beck Depression Inventory (BDI), and pain scales in advanced cervical cancer patients. Health Care Women Int..

[38] Domhardt M (2021). Mediators and mechanisms of change in internet-and mobile-based interventions for depression: A systematic review. Clin. Psychol. Rev..

[39] Josephine K, Josefine L, Philipp D, David E, Harald B (2017). Internet-and mobile-based depression interventions for people with diagnosed depression: A systematic review and meta-analysis. J. Affect. Disord..

[40] Andersson G, Titov N, Dear BF, Rozental A, Carlbring P (2019). Internet-delivered psychological treatments: From innovation to implementation. World Psychiatry.

[41] Schulz KF, Altman DG, Moher D (2010). Research methods & reporting. Br. Med. J..

[42] American Psychiatric Association (2015). Structured Clinical Interview for DSM-5 (SCID-5).

[43] Furlanetto LM, Mendlowicz MV, Bueno JR (2005). The validity of the Beck depression inventory-short form as a screening and diagnostic instrument for moderate and severe depression in medical inpatients. J. Affect. Disord..

[44] Heisel MJ, Flett GL (2005). A psychometric analysis of the geriatric hopelessness scale (GHS): Towards improving assessment of the construct. J. Affect. Disord..

[45] Shelton, R. C. Selective serotonin reuptake inhibitors and related antidepressants. In *The American Psychiatric Association Publishing Textbook of Mood Disorders* 239 (2022).

[46] van der Vinne N (2021). EEG biomarker informed prescription of antidepressants in MDD: A feasibility trial. Eur. Neuropsychopharmacol..

[47] Mac Giollabhui N (2018). Negative cognitive style interacts with negative life events to predict first onset of a major depressive episode in adolescence via hopelessness. J. Abnorm. Psychol..

[48] Beck AT, Steer RA (1988). Manual for the Beck Hopelessness Scale.

[49] Beck AT, Weissman A, Lester D, Trexler L (1974). The measurement of pessimism: The hopelessness scale. J. Consult. Clin. Psychol..

[50] Dejkam N, Sharifi H, Human H (2003). Conformity and Norm of the Beck Hopelessness Scale Among Students of Tehran Islamic Azad University.

[51] Goudarzi, M. *The Study of Reliability and Validity of Beck Hopelessness Scale in a Group of Shiraz University Students* (2002).

[52] Beck AT, Beck RW (1972). Screening depressed patients in family practice: A rapid technic. Postgrad. Med..

[53] Beck, A. T., Steer, R. A. & Brown, G. Beck depression inventory–II. *Psychological Assessment* (1996).

[54] Rajabi, G. R. *Psychometric Properties of Beck Depression Inventory Short Form Items (BDI-13)* (2005).

[55] Miller IW, Norman WH, Bishop SB, Dow MG (1986). The modified scale for suicidal ideation: Reliability and validity. J. Consult. Clin. Psychol..

[56] Cochrane-Brink KA, Lofchy JS, Sakinofsky I (2000). Clinical rating scales in suicide risk assessment. Gen. Hosp. Psychiatry.

[57] Esfahani M, Hashemi Y, Alavi K (2015). Psychometric assessment of beck scale for suicidal ideation (BSSI) in general population in Tehran. Med. J. Islam Repub. Iran.

[58] Yennurajalingam S (2018). Cranial electrotherapy stimulation for the management of depression, anxiety, sleep disturbance, and pain in patients with advanced cancer: A preliminary study. J. Pain Sympt. Manag..

[59] Boß L (2016). Reliability and validity of assessing user satisfaction with web-based health interventions. J. Med. Internet Res..

[60] Shaygan M, Yazdani Z, Valibeygi A (2021). The effect of online multimedia psychoeducational interventions on the resilience and perceived stress of hospitalized patients with COVID-19: A pilot cluster randomized parallel-controlled trial. BMC Psychiatry.

[61] Vojdany S, Golzari M, Borjali A (2014). The Effectiveness of positive psychotherapy on depression and marital satisfaction of depressed women. J. Appl. Psychol..

[62] Cohen J (2013). Statistical Power Analysis for the Behavioral Sciences.

[63] Graneheim UH, Lundman B (2004). Qualitative content analysis in nursing research: Concepts, procedures and measures to achieve trustworthiness. Nurse Educ. Today.

[64] Lincoln, Y. & Guba, E. (CA USA, 1985).

[65] Hollon SD, Shelton RC, Loosen PT (1991). Cognitive therapy and pharmacotherapy for depression. J. Consult. Clin. Psychol..

[66] Cuijpers P (2013). A meta-analysis of cognitive-behavioural therapy for adult depression, alone and in comparison with other treatments. Can. J. Psychiatry.

[67] Golshan A, Zargham Hajebi M, Sobhi Gharamaleki N (2019). The effect of logotherapy group training on changes of depression, self-esteem and intimacy attitudes in physically disabled women. Iran. J. Health Psychol..

[68] Mahdizadeh M, Alavi M, Ghazavi Z (2016). The effect of education based on the main concepts of logotherapy approach on the quality of life in patients after coronary artery bypass grafting surgery. Iran. J. Nurs. Midwifery Res..

[69] Beyrami M, Osfoori M, Esfahani A (2016). Efficacy of group logo therapy on coping strategies with stress and adjustment to illness in leukemia patients. Iran. J. Psychiatr. Nurs..

[70] Baran Oladi S, Sheykhpoor N, Mortezavi SM, Sabahi A (2018). The effect of group logotherapy on hopeness in spinal cord injury patients. Knowl. Res. Appl. Psychol..

[71] Ghelbash Z, Yektatalab S, Momennasab M, Foruhi Z (2020). Effect of group-based logotherapy on imprisoned women’s level of hope: A randomized controlled trial (RCT). Int. J. Prison. Health.

[72] Kamae A, Weisani M, Sadatizadeh S (2014). Logo therapy effect on life expectancy & suicidal thoughts among women with AIDS, community of values revival. Indian J. Fundam. Appl. Life Sci..

[73] Pocock SJ (2013). Clinical Trials: A Practical Approach.

[74] Brody, D. J., Pratt, L. A. & Hughes, J. P. *Prevalence of Depression Among Adults Aged 20 and Over: United States, 2013–2016* (2018). 29638213

[75] Vos T (2017). Global, regional, and national incidence, prevalence, and years lived with disability for 328 diseases and injuries for 195 countries, 1990–2016: A systematic analysis for the Global Burden of Disease Study 2016. The Lancet.

[76] Gelenberg AJ (2010). American Psychiatric Association practice guidelines for the treatment of patients with major depressive disorder. Am. J. Psychiatry.

[77] Zisi V, Gratsani S, Leontari D, Theodorakis Y (2016). Combining individual and group counselling sessions in a smoking cessation intervention. Psychology.

[78] Bolhari J, Naziri G, Zamanian S (2012). Effectiveness of spiritual group therapy in reducing depression, anxiety, and stress of women with breast cancer. Q. J. Woman Soc..

[79] Firth J (2017). The efficacy of smartphone-based mental health interventions for depressive symptoms: A meta-analysis of randomized controlled trials. World Psychiatry.

[80] Linardon J, Cuijpers P, Carlbring P, Messer M, Fuller-Tyszkiewicz M (2019). The efficacy of app-supported smartphone interventions for mental health problems: A meta-analysis of randomized controlled trials. World Psychiatry.

[81] Donker T (2013). Smartphones for smarter delivery of mental health programs: A systematic review. J. Med. Internet Res..

[82] Naslund JA, Aschbrenner KA, Marsch LA, Bartels SJ (2016). The future of mental health care: Peer-to-peer support and social media. Epidemiol. Psychiatr. Sci..

[83] Ostic D (2021). Effects of social media use on psychological well-being: A mediated model. Front. Psychol..

[84] Carlbring P, Andersson G, Cuijpers P, Riper H, Hedman-Lagerlöf E (2018). Internet-based vs face-to-face cognitive behavior therapy for psychiatric and somatic disorders: An updated systematic review and meta-analysis. Cogn. Behav. Ther..

[85] Gupta C, Jogdand DS, Kumar M (2022). Reviewing the impact of social media on the mental health of adolescents and young adults. Cureus.

[86] Chen Y, Gao Q (2022). Effects of social media self-efficacy on informational use, loneliness, and self-esteem of older adults. Int. J. Hum. Comput. Interact..

[87] Karim F, Oyewande AA, Abdalla LF, Chaudhry Ehsanullah R, Khan S (2020). Social media use and its connection to mental health: A systematic review. Cureus.

[88] Berry N (2017). # WhyWeTweetMH: Understanding why people use Twitter to discuss mental health problems. J. Med. Internet Res..

[89] Firth J (2017). The efficacy of smartphone-based mental health interventions for depressive symptoms: A meta-analysis of randomized controlled trials. World Psychiatry.

[90] Marco JH, Pérez S, García-Alandete J (2016). Meaning in life buffers the association between risk factors for suicide and hopelessness in participants with mental disorders. J. Clin. Psychol..

[91] Bartrés-Faz D, Cattaneo G, Solana J, Tormos JM, Pascual-Leone A (2018). Meaning in life: Resilience beyond reserve. Alzheimer's Res. Ther..

[92] Batthyány A (2016). Logotherapy and Existential Analysis: Proceedings of the Viktor Frankl Institute Vienna.

[93] Costanza A, Prelati M, Pompili M (2019). The meaning in life in suicidal patients: The presence and the search for constructs. A systematic review. Medicina.

[94] Attoe AD, Chimakonam JO (2020). The COVID-19 pandemic and meaning in life. Phronimon.

[95] Liu ST (2020). Serial multiple mediation of demoralization and depression in the relationship between hopelessness and suicidal ideation. Psychooncology.

[96] Gravier AL (2020). Meaning in life in patients with advanced cancer: A multinational study. Support. Care Cancer.

