# Supplementary material for: The effect of mobile-based logotherapy on depression, suicidal ideation, and hopelessness in patients with major depressive disorder: a mixed-methods study
Source: Sci Rep. 2023 Sep 22;13:15828. doi: 10.1038/s41598-023-43051-8 (PMC10516998; doi:10.1038/s41598-023-43051-8)
Supplement: Supplementary file 1 — Supplementary Information. [file 41598_2023_43051_MOESM1_ESM.docx]

**Supplementary 1.** Kolmogorov-Smirnov Test Results for Data Distribution Normality Assessment

| **Variable** | **Assessment Time Points** | **Kolmogorov-Smirnov** | | |
| --- | --- | --- | --- | --- |
|  |  | **Statistic** | **Degrees of Freedom** | **Significance Level** |
| **Depression** | Pretreatment | .116 | 70 | 0.07 |
|  | Posttreatment | .103 | 70 | 0.06 |
|  | Follow-up | .078 | 70 | 0.20 |
| **Hopelessness** | Pretreatment | .148 | 70 | 0.07 |
|  | Posttreatment | .177 | 70 | 0.06 |
|  | Follow-up | .178 | 70 | 0.06 |
| **Suicidal ideation** | Pretreatment | .138 | 70 | 0.55 |
|  | Posttreatment | .183 | 70 | 0.53 |
|  | Follow-up | .223 | 70 | 0.58 |
